# From Criminal Liability to Patient Safety: The Possible Impact of the Italian 2025 Reform Proposal on Senior Healthcare Leadership and Clinical Risk Management

**DOI:** 10.3390/healthcare14111494

**Published:** 2026-05-28

**Authors:** Sandro La Micela, Gloria Stevanin, Anna Pancheri, Camilla Faes, Annamaria Bonetti, Silvia Atti, Ilaria Tocco Tussardi, Stefano Tardivo

**Affiliations:** 1 Healthcare Safety and Claims Management Service, Integrated University Healthcare Authority of Trentino, 38123 Trento, Italy; sandro.lamicela@asuit.tn.it (S.L.M.);; 2 Provincial Hospital Service Management, Integrated University Healthcare Authority of Trentino, 38123 Trento, Italy; 3 Hospital Medical Directorate, Hospital of Tione, Integrated University Healthcare Authority of Trentino, 38123 Trento, Italy; 4 Hospital Medical Directorate, Hospitals of Borgo Valsugana and Cavalese, Integrated University Healthcare Authority of Trentino, 38123 Trento, Italy; 5 Department of Diagnostics and Public Health, Section of Hygiene, University of Verona, 37134 Verona, Italy; 6 Risk Management, University Hospital of Verona, 37126 Verona, Italy

**Keywords:** medical malpractice, organizational risk, healthcare liability, patient safety, clinical governance, top-management accountability, Italian criminal liability, Article 590-sexies, Article 590-septies, Law No. 24/2017 (“Gelli-Bianco Law”), systemic approach to medical error

## Abstract

This article analyses the Italian Legislative Delegation Bill of 4 September 2025 (DDL 2025), which proposes the recontextualization of healthcare liability through the introduction of Article 590-septies into the Italian Criminal Code (c.p.) and the amendment of Article 590-sexies c.p. and of Articles 5 and 7 of the Gelli-Bianco Act (Law No. 24/2017). The study examines the extent to which the reform, if enacted, would produce a shift of criminal negligence liability from the individual frontline clinician towards the apex management figures of healthcare organizations—at both the corporate and hospital levels—and under what conditions such a shift would be compatible with the constitutional principle of personal criminal responsibility (Art. 27 Const.) and with the evidentiary criteria for criminally relevant omission. Adopting a doctrinal and jurisprudential analysis approach, the study formulates a falsifiable hypothesis, accompanied by four ex post verifiability indicators observable over a five-year time horizon following the possible entry into force of the provision. The analysis demonstrates how the DDL 2025 would recontextualize the notion of culpa—encompassing imperizia (lack of skill), negligenza (negligence), and imprudenza (imprudence), functionally comparable to forms of criminal negligence in common law systems—by linking fault assessment to contextual factors such as organizational deficiencies and resource scarcity. This approach would adopt a deflationary framework, establishing a distinction between avoidable human error and errors caused by systemic dysfunctions and foreshadowing a potential shift of liability towards apex management, who are required to ensure organizational models adequate to patient safety. This orientation, far from constituting a doctrinal novelty, would formalize ex lege a trajectory already established in civil and criminal case law of the Court of Cassation (Cass. No. 6386/2023, “Travaglino”), further intersecting with the administrative liability regime for organizations under Legislative Decree 231/2001. Significant interpretive challenges remain, related to the application of criminal liability criteria to the omissive conduct of healthcare managers, as well as to the contrasting international evidence on the behavioural effectiveness of medical liability reforms. The redefinition of top-management liability would therefore be configured not merely as a tool for the protection of the individual professional but as a derived constitutional guarantee of the right to health and the safety of care, pursued through formalized risk governance, the integration of incident reporting and organizational audit systems, the transition towards Enterprise Risk Management models, and the traceability of apex decision-making processes. Examples drawn from other European jurisdictions illustrate the heterogeneity of legal approaches to medical fault and frame the Italian proposal as a context-specific solution that nonetheless could contribute to the international debate on institutional and organizational accountability for patient safety.

## 1. Introduction

For over half a century, the evolution of the Italian regulatory framework on professional healthcare liability has represented one of the most persistent and complex legal challenges. This evolution constitutes a constant search for equilibrium between the fundamental constitutional right to health [1] and the need to safeguard the clinical independence of healthcare professionals, protecting them from a disproportionate risk of litigation following adverse clinical outcomes.

The Legislative Delegation Bill approved by the Council of Ministers on 4 September 2025—providing for the “Delegation to the Government in the matter of healthcare professions and provisions concerning the professional liability of healthcare practitioners” [2]—marks the latest stage in this legislative trajectory. It aims to strengthen the Italian National Health Service (Sistema Sanitario Nazionale, SSN) and redefine the criminal law interpretation of fault (colpa) in healthcare. The reform seeks to protect professionals while simultaneously safeguarding the principles of human dignity and the centrality of patients’ needs.

The historical trajectory has traversed several distinct phases over the last seven decades [3,4].

In the initial phase (approximately 1950–1970), the system evolved from a paternalistic approach—characterized by a culture of favouring medicine and deep deference towards the medical profession—towards an approach that progressively recognized patients as holders of fully enforceable rights [1]. In the absence of sector-specific statutory provisions, criminal judges initially applied by analogy the criterion set out in in Article 2236 of the Civil Code [5]. This provision—designed for the civil liability of intellectual service providers—limited liability for lack of skill (imperizia) to cases of intent or gross negligence when problems of special technical difficulty were involved.

However, the law soon affirmed its autonomy from this civil law model, as confirmed by the judgment of the Constitutional Court No. 166 of 1973 [6]. The Court held that the protection of life and physical integrity could not be subordinated to the compensatory logic of civil law, emphasizing the need to move beyond the principle of “gross negligence” as the primary shield for the conduct of healthcare professionals.

The subsequent phase (late 1970s–1980s) confirmed this shift and was characterized by heightened attention to patient rights and a consequent stringency in liability standards, leading to the undifferentiated application of ordinary evaluative criteria to healthcare conduct. This period witnessed a shift from probabilistic standards towards “possibilistic” concepts, in which any deviation from the required standard of care could give rise to criminal proceedings. This interpretative rigidity, coupled with rising societal expectations and fear of litigation, fostered defensive medicine: an attitude in which clinical decisions were guided by the need for “unassailable” documentation rather than by clinical necessity. This resulted in the over-prescription of diagnostic investigations and unnecessary treatments, driving practitioners to avoid high-risk procedures or to refuse complex patient cases.

To address these systemic dysfunctions, the case law of the 2000s sought new parameters to circumscribe criminal proceedings. A landmark development was the “Franzese” ruling by the United Sections of the Court of Cassation (Criminal United Sections, No. 30328/2002) [7]. Without modifying the substantive definition of fault, it established a rigorous and definitive criterion for the determination of the causal nexus. It required that, for a conviction to stand, the aetiological link between the professional’s conduct (whether active or omissive) and the adverse event had to be proven with a high degree of logical probability, satisfying the standard of “beyond reasonable doubt”.

Parallel to this, the use of clinical guidelines and best practices began to be recognized as soft law and objective benchmarks for evaluating professional conduct. However, the absence of statutory provisions governing the legal value of such guidelines led to profound jurisprudential inconsistencies in the decade preceding 2012, with conflicting orientations on the implications of compliance with or deviation from such recommendations [8].

Decree-Law No. 158/2012 (converted into Law No. 189/2012, known as the “Balduzzi Decree”) [9] attempted to formalize the role of clinical guidelines and reintroduced the distinction between slight and gross negligence in criminal proceedings. It granted immunity for slight negligence to practitioners who adhered to accredited guidelines and best practices. Despite its intentions, it failed to resolve pre-existing uncertainties; on the contrary, it generated jurisprudential debate regarding the absence of a clear definition of “minor negligence” and ambiguity as to which guidelines were to be considered “accredited” [10,11].

Only five years later, Law No. 24 of 8 March 2017 (the so-called “Gelli-Bianco Law”) [12], concluding a lengthy and troubled legislative process, introduced the concept of “safety of care” (Art. 1) as a constitutive element of the right to health. In terms of criminal liability, the law repealed the provision of the Balduzzi Decree and introduced Article 590-sexies of the Italian Criminal Code (c.p.—codice penale) [13]. This article limited the exclusion of punishability to cases of lack of skill (imperizia) only, provided that the guidelines appropriate to the specific case had been observed (or, in their absence, good clinical and care practices). Negligence (negligenza) and imprudence (imprudenza) remained punishable.

Despite its aims, the Gelli-Bianco Law proved interpretatively problematic as well [14]. The subsequent “Mariotti” judgment (Joint Criminal Divisions of the Court of Cassation, No. 8770/2018) [15] further specified the scope of Art. 590-sexies, limiting non-punishability to cases of minor imperizia occurring in the executive phase of the medical act, provided that appropriate guidelines had been followed. Diagnostic errors, errors in therapeutic choice, or conduct characterized by negligence or imprudence—even if minor—remained prosecutable [16,17].

Despite the framework of Law 24/2017 and the extensive jurisprudence that followed, the search for a definitive equilibrium remains ongoing. The 2025 Legislative Delegation Bill proposes further amendments to the Criminal Code and the Gelli-Bianco Law, with the goal of containing defensive medicine and strengthening the protection of healthcare professionals operating in an increasingly resource-scarce and organizationally complex system.

The Italian normative trajectory described above intersects with a broader international debate on the determinants of patient safety. International literature has long emphasized that adverse events in healthcare are predominantly the result of system-level failures rather than individual professional errors and that effective safety policies require organizational, cultural, and managerial interventions that go beyond the regulation of individual professional conduct [18,19,20,21].

In parallel, several jurisdictions have debated whether legal reform aimed at reducing individual professional liability can effectively constitute a brake on defensive medicine while simultaneously improving systemic accountability, with contrasting empirical results [22,23,24].

In this context, the Italian 2025 reform proposal may be interpreted as an attempt to translate, within a national legal framework, the international consensus on the systemic approach to patient safety, while preserving the personal nature of criminal liability under Article 27 of the Italian Constitution [25].

This article offers a critical analysis of the proposed normative framework, seeking to answer to what extent the proposed reform would produce, if enacted, a shift of criminal negligence liability in the healthcare sector from the individual frontline clinician towards the apex management figures of healthcare organizations and under what conditions such a shift would be compatible with the principle of personal criminal responsibility and with the evidentiary criteria for criminally relevant omission [25].

## 2. Materials and Methods

This study adopts a doctrinal and jurisprudential analysis (doctrinal–jurisprudential analysis) of the Italian normative framework governing healthcare liability and recent legislative reform proposals. Italian legal sources were selected according to a formal hierarchy: (i) constitutional sources (Constitution, Art. 32); (ii) primary sources (Criminal Code, in particular Arts. 590-sexies and the proposed Art. 590-septies; Civil Code, Art. 2236; Legislative Decree 502/1992; Legislative Decree 231/2001; Law 189/2012; Law 24/2017; and Legislative Delegation Bill of 4 September 2025); (iii) legality case law (Constitutional Court and Court of Cassation, criminal and civil divisions, with priority given to Joint Divisions rulings and judgments recognized as leading cases in the period 2002–2024); (iv) specialist medico-legal and criminal law doctrine.

The inclusion criteria for case law were: (a) relevance for the characterization of medical fault (culpa medica) and for the identification of the omissive causal nexus; (b) relevance for the position of guarantee of apex healthcare management figures; (c) relevance for the characterization of organizational fault (colpa di organizzazione). The temporal scope considered is 1973–2024, with primary focus on the period 2002–2024 (post-Franzese judgment).

The potential impact of the proposed legislative amendments is ultimately assessed from both a medico-legal and an organizational perspective within healthcare structures. To this end, a detailed analysis examines the professional profiles of the apex managers of public healthcare organizations—namely the Chief Executive Officer (Direttore Generale, DG), the Chief Medical Officer (Direttore Sanitario, DS), and the Hospital Medical Director (Direttore Medico di Presidio, DMP)—with particular attention to their respective management and supervisory functions and how the possible redefinition of apex criminal liability may represent a stimulus for further organizational improvement and with evident positive repercussions on the safety of care.

It is expressly noted that the considerations developed in this work are grounded in the analysis of a bill which, at the present time, has not yet acquired normative force. Consequently, every interpretive or applicative assessment relating to this proposal must be understood in necessarily hypothetical terms and subject to revision in light of the potential evolution of the legislative process and the subsequent jurisprudential elaboration of the courts in the years following its possible entry into force.

To enhance terminological consistency and support the interpretation of context-specific concepts, a table containing the operational definitions of the principal Italian legal terms adopted in this study is provided (Table 1).

## 3. Integrated Analysis and Potential Scenarios

The Legislative Delegation Bill approved by the Council of Ministers on 4 September 2025 [2] introduces innovations in both criminal and civil law, signalling a paradigm shift from previous regulatory frameworks. Specifically, the proposal includes amendments to Article 590-sexies of the Criminal Code, the introduction of a new Article 590-septies, and modifications to Articles 5 and 7 of the Gelli-Bianco Law (No. 24/2017) [14].

The revised Article 590-sexies c.p. [13] seeks to reinstate the principle of non-punishment for slight negligence on a broader scale. This moves beyond the restricted focus on “lack of skill” established by the Gelli-Bianco Law. Specifically, this amendment aims to extend the exemption to all forms of fault—negligence, imprudence, and lack of skill—provided they are classified as “minor” and occurred in the context of conduct compliant with guidelines appropriate to the specific features of the case [2].

The new Article 590-septies c.p. formalizes the contextualization of criminal fault. It links judicial assessment not only to the practitioner’s actions but also to operational context and systemic constraints. The provision delineates a series of objective and contextual parameters that must be considered when ascertaining fault or its degree, including: a scarcity of human and material resources or structural inadequacies beyond the practitioner’s control; the absence, limitation, or contradictory nature of scientific knowledge regarding the pathology or treatment; the actual availability of appropriate therapies; the complexity of the pathology or the inherent difficulty of the medical act; the specific role performed within multidisciplinary cooperation; and the presence of urgent or emergency situations [2].

Regarding Law 24/2017 [14], the Legislative Delegation Bill provides further clarifications within the civil domain. Concerning Article 5, it specifies that the application of clinical and care best practices (utilized in the absence of formal guidelines) must be evaluated against the specificity of the concrete case [2].

Furthermore, the introduction of paragraph 3-bis to Article 7 extends the evaluative criteria of Article 590-septies c.p. to civil proceedings. It mandates that courts account for contextual factors such as scarcity of human and material resources and organizational shortcomings, explicitly invoking the principles of Article 2236 of the Civil Code.

The 2025 Bill represents a significant attempt to provide legal certainty by steering jurisprudence toward a deeper understanding of the operational and organizational constraints influencing medical acts.

This approach distinguishes between avoidable human error and errors caused by organizational deficiencies, reconfiguring the determination of liability on two distinct and complementary levels: the possible decriminalization of the frontline practitioner’s fault, where the event is caused by organizational shortcomings, finds its necessary counterweight in the greater weight attributed, for the purposes of criminal imputation, to the omissions of those who, by institutional mandate, hold a systemic guarantor duty with respect to resources, organization and governance.

At a practical level, the reform paves the way for the identification of the specific natural persons on whom legal responsibility for the structural shortcomings that caused the adverse event may fall: the apex management figures—Chief Executive Officers, Chief Medical Officers and Hospital Medical Directors—who are directly responsible for managerial decisions, resource allocation and the implementation of organizational measures aimed at preventing systemic deficiencies.

This distinction would also carry significant implications for the allocation of the evidentiary and legal burden: where an adverse event cannot be entirely attributed to the conduct of an individual clinician, but instead reflects deeper systemic failures, the proposed framework would open the way for identifying those specific individuals—CEOs, CMOs and HMDs—who bear legal responsibility for such structural inadequacies by virtue of their decision-making power and institutional duty. If enacted, the reform would therefore operate on two complementary levels: on the one hand, shielding frontline practitioners from criminal liability for minor fault causally linked to organizational deficiencies beyond their control; on the other, extending the reach of culpable omission to those apex managers who held both the duty and the concrete power to prevent those failures.

### 3.1. Working Hypothesis and Verifiability Indicators

On the basis of the normative analysis developed in the preceding section, this work formulates the hypothesis that the complex of amendments introduced by the 2025 Legislative Delegation Bill would produce a measurable expansion of the subjects involved in criminal negligence liability in the healthcare sector. Such expansion would be expressed not through a generalized redistribution of imputability but through the subjective requalification of organizational fault attributable to the apex management figures of healthcare organizations in cases where the adverse event is aetiologically attributable, on a causal or co-causal basis, to structural, organizational, or governance deficiencies.

The hypothesis may be verified, in principle, through the systematic observation—over a five-year period following the potential entry into force of the legislation—of a set of indicators that should be understood as a working proposal rather than a definitive reference framework. These indicators stem from a reflection we undertook regarding which measurable parameters might capture the concrete effects of the law on the criminal liability of apex management figures, should it be enacted.

The indicators we propose concern: (i) changes in the number of criminal proceedings initiated against top-level managerial figures in the healthcare sector pursuant to Articles 589 and 590 of the Italian Criminal Code; (ii) qualitative shifts in the nature of the charges formulated in proceedings related to adverse events, including allegations of organizational negligence; (iii) the ratio between convictions and case dismissals in proceedings involving apex management figures; (iv) measurable changes in corporate risk governance, encompassing the formal restructuring of the clinical risk management function within organizational charts, the volume of documented organizational audits, and the proportion of budget specifically allocated to clinical risk management activities.

The intrinsic limitations of an analysis conducted in the pre-enactment phase are expressly acknowledged: the not yet prescriptive character of the Legislative Delegation Bill; the margin for modification of the text during the parliamentary process; and the impossibility of measuring ex ante the indicators listed above. The proposed indicators therefore constitute an ex post observation framework, intended for the future empirical validation or falsification of the hypothesis, whose implementation remains conditional on the actual conclusion of the legislative process.

### 3.2. Apex Healthcare Management: Roles and Responsibilities

This emerging legal framework creates significant challenges in determining individual and organizational responsibility. Public healthcare organizations exhibit structural variability influenced by diverse regional policy directives. Nevertheless, Article 3, paragraph 1-quater, of Legislative Decree No. 502/1992 defines the governing bodies of a public healthcare organization [26]. These include the Chief Executive Officer (CEO), supported by the Chief Administrative Officer (CAO), the Chief Medical Officer (CMO), the medical council, the executive board, and the board of statutory auditors.

#### 3.2.1. The Chief Executive Officer (CEO)

The CEO serves as the legal representative and head of the public healthcare organization, holding supreme executive and representational authority. Specifically, the CEO

- Adopts the corporate governance act under private law, ensuring compliance with the principles of regional (or provincial) programming;
- Bears overall responsibility for corporate management;
- Appoints the Chief Medical Officer and the Chief Administrative Officer, who assist in achieving the strategic objectives (thereby constituting the strategic management);
- Issues regulatory acts and adopts the relevant interpretive and applicative guidelines;
- Defines objectives, priorities, plans, programmes, and directives for institutional and management activities;
- Allocates human, material, and financial resources;
- Oversees the proper management of resources;
- Ensures the impartiality and efficiency of administrative action;
- Is appointed by the region or province under a fixed-term contract and may be removed for failure to achieve objectives [26].

#### 3.2.2. The Chief Medical Officer (CMO)

The CMO, alongside the CAO, assists the CEO in institutional management through proposals and expert opinions. The CMO assumes direct professional responsibility for functions within their clinical purview. Furthermore, the CMO manages healthcare services to ensure compliance with organizational, sanitary, and public health standards. Specifically, the CMO

- Participates in the strategic management of the organization;
- Assumes direct professional responsibility within the specific scope of his or her competences;
- Contributes to the CEO’s decisions of a scientific nature by formulating proposals and technical opinions [26].

#### 3.2.3. The Hospital Medical Director (HMD)

Within public hospital facilities, the Hospital Medical Director plays a pivotal role. This figure

- Operates in compliance with the directives issued by the Chief Medical Officer;
- Ensures continuity of care with territorial Healthcare Districts;
- Holds managerial, organizational, sanitary-hygiene, medico-legal, scientific, and training responsibilities;
- Promotes the quality of healthcare services and clinical performance;
- Is responsible for the clinical governance of the hospital;
- Promotes initiatives aimed at coordinating and improving the efficiency, effectiveness, and appropriateness of healthcare services;
- Manages, coordinates, supports, and supervises the medical directors of complex, departmental, and simple operative units, fostering multidisciplinary integration both within the hospital and with territorial services [26].

The mapping of the responsibilities of the management figures described above must always be contextualized against the background that resource allocation, staffing, and fund allocation are constrained by the spending ceilings imposed by regional or provincial programming and by the limits of the national health fund. The criminal negligence of an apex manager, within the framework of the proposed Art. 590-septies c.p., must be determined in terms of violation of specific duties, foreseeable and exigible in the concrete operational context of the organization.

Among the duties listed above, some are characterized by greater potential exposure under the proposed Art. 590-septies c.p., as they bear directly on the contextual parameters of the provision. In particular: (i) human resource allocation and staffing planning (CEO, CMO) correlate directly to the parameter of “scarcity of human resources”; (ii) planning and management of healthcare technologies (CMO, HMD) correlates to “scarcity of material resources”; (iii) the promotion of clinical governance, multidisciplinary integration, and safety protocols correlates to “organizational deficiencies” and “multidisciplinary cooperation”; (iv) continuing professional development and personnel training correlates to the adequacy of available “scientific knowledge” (v) the preparation and updating of emergency management plans correlates to “emergency or urgency situations”.

## 4. Personal Liability and Normative Frameworks

These activities entail substantial responsibilities. Executive figures may incur legal liability, including criminal charges, when professional negligence or non-compliance is established.

The practical implications of the Legislative Delegation Bill of 4 September 2025 and current regulations require careful analysis. The roles of apex management figures must be clearly defined with regard to their specific personal responsibility.

Under the proposed legislative changes, the assessment of individual healthcare professionals’ liability must take into account specific omissions by executive leadership. If governance deficits give rise to a legally relevant adverse event, responsibility (both civil and criminal) may extend to apex managers within their respective areas of competence.

Organizational responsibility of institutional and facility executives encompasses various scenarios, ranging from public health issues to broader management concerns, defined as “contextual factors” by the new legislation. Under this regulatory framework, executives may be held responsible in multiple situations based on their respective areas of responsibility:

- Human Resources Management: Managers must ensure adequate staffing planning through systematic needs assessment. This includes the timely transmission of staffing requests to strategic management and Human Resources, the initiation of authorization procedures with regional or provincial funding bodies, the monitoring of workload sustainability, and the adoption of measures to ensure continuity of care in cases of staffing shortages.
- Healthcare Technology Planning and Management: Managers must develop a strategic plan for the acquisition, replacement, and maintenance of biomedical technologies, in collaboration with Clinical Engineering. Such planning must be based on a systematic assessment of equipment obsolescence, the implementation of Health Technology Assessment (HTA) processes for new technologies, the analysis of the health needs of the target population, and the prioritization of investments consistent with health objectives and available resources.
- Pharmaceutical and Medical Device Supply Chain Management: Managers must ensure the timely and appropriate availability of pharmaceuticals and medical devices. Responsibilities include verifying the functionality of procurement systems, monitoring stocks to prevent shortages, ensuring compliance with pharmacovigilance and traceability protocols, and implementing quality control systems for medical devices.
- Continuing Medical Education (CME) Governance: Managers must ensure that healthcare personnel meet mandatory training requirements. They must guarantee equitable access to CME and professional development programmes, with particular attention to non-employed personnel (self-employed practitioners and consultants) to ensure uniform standards of clinical competence and patient safety, regardless of contractual status.
- Emergency and Critical Event Management: Managers must ensure the preparation, regular updating, broad dissemination, and operational knowledge of emergency plans by all personnel. These include: protocols for emergency department overcrowding and mass casualty incidents; Internal Mass Emergency Plans; Internal Emergency and Evacuation Plans; and specific Safety Plans for critical areas. Responsibility encompasses the organization of periodic drills, the verification of plan effectiveness, and the definition of escalation procedures during crises.

Consequently, apex managers may incur responsibility for legally relevant adverse events causally linked to systemic deficiencies.

In cases of chronic understaffing or technological obsolescence, responsibility may fall upon the executive responsible for resource planning [27]. Managers may be held accountable even where, despite being aware of persistent organizational deficiencies, they have failed to implement alternative organizational risk mitigation strategies.

Similarly, adverse clinical outcomes may result from outdated scientific knowledge (due to inadequate institutional training), insufficient protocols, or a lack of multidisciplinary integration. Where such deficiencies stem from managerial or organizational failures, the individual responsible for promoting efficiency and minimum clinical safety standards may be held liable. Therefore, apex managers bear responsibility for systemic deficiencies, including both failure to secure adequate resources and tolerance of organizational risk. In any case, the extension of criminal liability of healthcare managers in relation to systemic failures cannot be based on a mere automatic correlation between organizational shortcomings and individual fault. In this hypothetical future scenario, any expansion of the criminal liability of apex management figures will require limiting principles that prevent every organizational inefficiency from becoming a source of culpability, and instead reconstruct fault along the threefold axis of eligibility, foreseeability, and avoidability, in line with the general principles of criminal liability: the required conduct must be concretely exigible in the given context; the adverse event must be foreseeable as a causally relevant consequence of the omission; and the omitted action must have a high logical probability of preventing the harm.

## 5. The Current Legal Framework

The hypothesized liability of apex management figures arising from governance deficits is by no means an abstract novelty. On the contrary, it represents the legislative formalization of established jurisprudential trends that have already emerged through judicial interpretation in both criminal and civil jurisdictions.

### 5.1. Entity Liability Under Legislative Decree 231/2001: Points of Intersection

The considerations developed concerning the individual criminal liability of apex managers intersect with the distinct, yet complementary, regime of administrative liability of organizations provided for by Legislative Decree 231/2001 [28]. This legislation—originally conceived for offences against the public administration and progressively extended to include, among others, culpable manslaughter and serious culpable bodily harm committed in violation of regulations on accident prevention and occupational health (Art. 25-septies)—establishes an administrative liability arising from the commission of offences in the interest or for the benefit of the organization. The application of Legislative Decree 231/2001 to public healthcare organizations has been the subject of intense doctrinal and jurisprudential debate, with conflicting positions as to whether such entities fall within the concept of “entity” relevant for the purposes of the provision.

Regardless of the outcome of this debate, the suitability of the organization and management model provided for by Art. 6 of Legislative Decree 231/2001—as an exempting instrument in the event of an offence committed by an apex individual—stands in evident conceptual continuity with the criteria of organizational adequacy that the proposed Art. 590-septies c.p. would valorise for purposes of individual fault determination. An organizational framework compliant with the criteria of Legislative Decree 231/2001—documented, monitored, and traceable—would therefore constitute, even within the proposed reform framework, a substantial evidentiary element in favour of the strategic management in individual criminal proceedings, as well as a corporate risk prevention instrument.

### 5.2. Civil Liability and the “Travaglino” Judgment

In civil proceedings, the organizational aspects of healthcare structures received a decisive impetus from the Third Section of the Court of Cassation through judgment No. 6386/2023 (the “Travaglino” judgment) [29]. The Court addressed a case of healthcare-associated infection (HAI) resulting in death, in which widespread deficiencies in hospital-level infection prevention and control systems were alleged. The Court reaffirmed that the healthcare organization is liable for nosocomial infections arising during hospitalization when it is unable to demonstrate that it has adopted all appropriate and up-to-date preventive measures, thereby emphasizing the central role of organizational obligations and the regulation of the burden of proof. This landmark judgment provided an exhaustive catalogue of preventive measures, introducing specific evidentiary burdens required to attest that such measures had been effectively developed, adopted, and monitored and identifying the specific management figures bearing the corresponding guarantee obligations.

Although confined to the civil dimension of liability, without directly addressing the criminal liability of apex figures, the judgment marked a crucial turning point. Fault is no longer understood as an undifferentiated imputation to the institution as such; it may instead be attributed subjectively to the apex managers burdened with specific prevention and supervisory obligations. It follows that the institution’s liability is apportioned according to the specific omissions of each management figure in ensuring the managerial and sanitary-hygiene conditions necessary to prevent adverse events [29].

### 5.3. Ministerial Decree No. 232/2023 as a Possible Implementing Tool of Law No. 24/2017 and Benchmark of Organizational Adequacy

Ministerial Decree No. 232/2023 of 15 December 2023 [30], as an implementing instrument of Law No. 24/2017, has assumed importance not only in the quantification of risk reserves and claims provisions but also as a parameter of adequacy of the corporate organizational structure in the area of safety of care. Its failure or incomplete transposition by the strategic management, especially in the absence of processes for the identification, analysis, monitoring, and mitigation of clinical risk, could characterize elements of organizational fault with possible criminal relevance. The integration of different categories of risk—clinical, operational, financial, strategic, technological, legal, and reputational—now structurally characterizes modern healthcare organizations and is progressively leading to the overcoming of the reactive approach typical of traditional risk management, in favour of an integrated proactive governance system implemented through Enterprise Risk Management programmes [31]. In Italy, the Lombardy Region has formally initiated this transition with the approval of the Healthcare Enterprise Risk Management (HERM) model pursuant to Welfare Directorate-General Decree No. 20638 of 21 December 2023 [32], documented in the literature as the first Italian experience of systematic ERM in healthcare [33]. A parallel Italian line of research [34] is extending the application of ERM principles to the mitigation of risks associated with the introduction of artificial intelligence in healthcare processes.

### 5.4. Criminal Liability: Jurisprudential Precedents

In criminal law, while media and procedural attention typically focus on frontline practitioners, high-court jurisprudence has delineated a precise guarantor position for institutional management facing resource scarcity or systemic dysfunction.

Indeed, criminal proceedings have already been initiated to ascertain the liability of CEOs, CMOs (Chief Medical Officers), Hospital Medical Directors (HMDs), and other apex management figures whenever a harmful event resulted from manifest structural failures, chronic staffing shortages, failure to provide essential medical supplies, or the inadequacy of operational protocols.

Doctrine and case law have progressively valorised the concept of organizational fault (colpa di organizzazione)—a category that describes forms of liability in which the harmful event does not derive from a single individually identifiable act of negligence but from structural, procedural, or governance deficits attributable to the organization as a whole—originally developed in connection with the crime prevention models of Legislative Decree 231/2001 [35].

In this perspective, the obligation, enshrined in Art. 2086 of the Civil Code [36], to establish an organizational, administrative, and accounting structure adequate to the nature and size of the enterprise assumes particular importance especially for healthcare structures, in which organizational deficiencies may constitute a violation of precautionary rules and, in the presence of a causal nexus with the harmful event, give rise to civil and criminal liability on the part of the apex individuals responsible for preventing them [37,38,39].

Consequently, the absence or incomplete presence of an adequate organizational structure, as well as the absence of effective monitoring of its application, efficiency, and results, may constitute an index of organizational fault even in criminal terms, where the adverse event can in the specific case be traced back to such systemic deficiencies.

In the judgment of the Court of Cassation, Criminal Section, No. 4981/2004 [40], a fatal accident occurring in a hyperbaric chamber as the result of a fire is examined, recognizing the criminal liability of the medical director for failing to ensure adequate staffing and safety measures in a high-risk technological context. The decision focuses on the director’s position of guarantee with respect to the structural and organizational conditions required for the protection of patients and workers.

By contrast, the judgment of the Court of Cassation, Criminal Section, No. 18334/2018 [41], clarifies the limits of apex criminal liability, affirming that the head of a department cannot be held responsible for adverse events caused by the conduct of subordinate physicians where he or she has correctly fulfilled his or her duties of organization, management, coordination, and supervision. While reaffirming the principle of the personal nature of criminal liability, the judgment confirms that the correct performance of managerial functions leads to a demonstration of the absence of organizational liability.

The judgment of the Court of Cassation, Criminal Section, No. 32477/2019 [42], concerns the death of a patient in a private care home, in which the Court attributes liability to the medical director for “organizational fault” (colpa di organizzazione), emphasizing that the director is the ultimate guarantor of the quality of care and personnel coordination. Criminal liability may arise when the absence of protocols for patient admission, risk management, communication, and emergency response contributes causally to the adverse event [43].

Furthermore, the judgment of the Court of Cassation, Criminal Section, No. 50619/2019 [44], affirms that the manager who delegates assigned duties does not entirely lose his or her position of guarantee and may be held criminally liable where it is demonstrated that he or she failed to issue adequate instructions, to correctly organize the work of collaborators, or to verify compliance with organizational criteria [45].

The jurisprudential and normative evolution highlighted appears to anticipate the direction taken by the 2025 bill, which for the first time brings to the fore the issue of organizational risk that, while on the one hand may represent a ground for exemption for the clinician, on the other cannot shift the court’s attention away from the duties of apex figures in the area of patient safety and clinical risk management.

The reform trajectory of the 2025 Legislative Delegation Bill aligns structurally with this trend. The proposed introduction of Article 590-septies c.p. appears destined to crystallize the concept of “organizational fault”. This shifts a broader, objective concept—traditionally confined to civil litigation—toward a more pronounced subjective accountability within the institutional hierarchy.

Where a harmful event arises from organizational inadequacy, criminal liability may ascend the managerial chain of command. Executives will no longer be able to plead ignorance regarding practices or deficiencies that they had a legal duty to govern, prevent, and control. Consequently, organizational risk transitions from being a mere mitigating circumstance for the practitioner to becoming the foundational element of negligence for management. This shift is further bolstered by Article 1 of Law 24/2017, which elevates the safety of care to a constitutional rank as an integral part of the right to health. Apex management figures could thus be charged with a direct violation of this right—not merely as culpa in vigilando but as direct personal negligence for the omission of functional acts essential to constitutionally guaranteed safety.

This shift is further supported by Art. 1 of Law 24/2017, which expressly qualifies the safety of care as an integral part of the right to health constitutionally protected by Art. 32 of the Constitution, affirming that it is achieved also through the totality of activities aimed at the prevention and management of risk associated with the delivery of healthcare services and the appropriate use of structural, technological, and organizational resources.

The transition from normative theory to judicial practice will nonetheless pose significant interpretive complexities. Unlike civil proceedings, where the healthcare organization bears the burden of proving exact compliance within the contractual relationship with the patient, in criminal proceedings the constitutional presumption of innocence prevails and the burden of proof rests entirely with the prosecution. For a Chief Medical Officer or a Hospital Medical Director to be held criminally liable, it is not sufficient to establish a generic state of disorganization.

The evidentiary criterion enunciated by the Joint Divisions of the Court of Cassation with the Franzese judgment (Cass. Joint Divisions No. 30328/2002) in the omissive scenario of the healthcare manager remains a complex operation. For the purpose of establishing the causal nexus in culpable omissive offences, a twofold verification is required: (i) proof, according to the criterion of high logical probability, that the omitted obligatory conduct, if carried out, would have prevented the event; (ii) counterfactual verification of the exclusion, beyond reasonable doubt, of alternative causal pathways.

While it is true that criminal case law has already concretely applied these principles to omissive managerial fault, one cannot deny the practical difficulty in distinguishing, in the specific case, the criminal relevance of the various responsibilities, especially where the adverse event is generated by the interaction of a plurality of systemic deficiencies extending to the possible fault of the individual practitioner.

It should be noted that the orientation described is not free from doctrinal and strictly operational objections. First, the possible expansion of the area of criminal liability to apex managers risks generating applicative uncertainty in a legal domain where the importance of the burden of proof is particularly relevant to procedural sustainability. Second, there is a risk that systemic responsibility will always be invoked as an indemnity shield for the individual practitioner’s fault. Third, the proof of the causal nexus between the organizational omission and the adverse event, in the light of the stringent criminal evidentiary criteria for the determination of fault, presents technical difficulties of complex resolution.

It is furthermore appropriate to maintain prudence in projections regarding the behavioural effectiveness of the reform. International literature on the impact of medical liability reforms on clinical behaviour and defensive medicine shows contrasting results: studies conducted in different contexts [22,23,24] document that the modification of the individual liability regime alone does not automatically translate into a measurable reduction of defensive medicine or a systemic improvement of safety in the absence of parallel interventions on organizational culture, training, and error learning systems—which require a complementary investment in risk governance, managerial training, and institutional accountability systems.

### 5.5. Broader Relevance and Transferability of the Italian Reform Model

The analysis carried out here is, by methodological choice, focused on a single national legal system; the trajectory it describes, however—the progressive shift from the fault of the individual practitioner towards organizational liability as a possible locus of imputation in the healthcare domain—reflects a tension that is not exclusively Italian. International literature has consistently documented that adverse events in healthcare are often systemic in origin rather than attributable to the isolated error of a single healthcare professional alone [18,19,20,21].

This notion has been strongly endorsed by the OECD as well, which has emphasized the need to move beyond a punitive model centred on individual fault (the “person approach”) in favour of a “just culture”, in which the accountability of healthcare organizations occupies a central role. In this regard, the OECD has reported that all 25 countries responding to the 2019 Survey of Patient Safety Governance had already adopted dedicated legislation for the promotion of patient safety. The legal instruments through which this premise has been translated into actionable accountability mechanisms differ substantially across jurisdictions, however, partly because they are shaped by the broader governance models of the individual national systems [46].

France, with Loi n° 2002-303 of 4 March 2002 (the “Loi Kouchner”), introduced a hybrid compensation pathway in which no-fault indemnification of therapeutic hazard (aléa thérapeutique) under solidarité nationale, administered through ONIAM, coexists with an amicable, non-binding conciliation procedure before the regional Commissions de Conciliation et d’Indemnisation; in order to analyse compensation data and identify the most preventable incidents, an Observatory of Medical Risks (Observatoire des risques médicaux) was subsequently established in 2005 [47]. In Germany, the Patientenrechtegesetz of 26 February 2013 codified the treatment contract within the Bürgerliches Gesetzbuch, introducing specific documentation duties and reversing the burden of proof in cases of fully manageable risk and gross treatment error; the systemic quality and risk management obligations binding on hospitals remain regulated separately [48,49]. In the United Kingdom, following the 2013 Francis Report on the serious failures of the Mid-Staffordshire NHS Trust, Regulation 20 of the Health and Social Care Act 2008 (Regulated Activities) Regulations 2014 introduced a statutory organizational duty of candour, binding on registered providers as institutions and operating in parallel with the professional duties imposed on individual clinicians by the General Medical Council and the Nursing and Midwifery Council [50]. Lastly, Sweden, Denmark, Norway and Finland have for decades operated patient-injury compensation schemes described as “no-fault”, resting on an objective criterion of avoidability that decouples individual compensation from the proof of fault; part of the literature notes that such schemes operate alongside institutional mechanisms oriented towards incident reporting and learning (the “learning approach”), although the actual strength of the preventive and accountability effects typical of fault-based liability continues to raise debate and concern [51].

These divergent solutions share a common analytical premise—the predominantly systemic origin of adverse events—but differ substantially in the legal instrument adopted to render liability operational, including in its organizational dimension. What distinguishes the Italian reform proposal is its possible codification, within the criminal law domain, of organizational deficiency as a condition limiting the liability of the individual practitioner—and, in so doing, opening the way to the introduction of organizational fault—rather than through no-fault compensation systems or forms of regulatory liability of healthcare entities. This specificity reflects features distinctive of the Italian system—including the constitutional principle of the personal nature of criminal liability (Art. 27 Cost.), the doctrine of the guarantor position (posizione di garanzia), and the evidentiary criterion of high logical probability established by the Franzese judgment—and is therefore difficult to transpose to different legal systems. The underlying analytical approach nevertheless retains a broader relevance: the identification of apex management figures as institutional bearers of legal duties with respect to organizational conditions. By directly affecting patient safety outcomes, such figures engage with the international debates currently underway on clinical governance and institutional accountability in healthcare. From this perspective, the Italian proposal is best interpreted not as a legislative template to be replicated but as a starting point for scholarly and legal reflection on how to translate systems thinking about patient safety into actionable institutional obligations.

## 6. Conclusions

The formulation of the new Article 590-septies of the Italian Criminal Code, as proposed by the 2025 legislative delegation bill, would—if enacted—represent the first piece of legislation to formalize an emerging jurisprudential trend, conferring for the first time formal statutory recognition upon organizationally grounded criminal negligence.

Although the legislative process remains ongoing, and further amendments in the course of parliamentary debate cannot be excluded, the bill’s formulation already offers a valuable opportunity for in-depth technical analysis. The possible evolution of this provision, regardless of its ultimate adoption, demands a no longer passive role from scientific and legal communities. Without waiting for the issuance of implementing decrees, stakeholders would need to engage with the innovative criteria underpinning the reform in order to anticipate its effects and contribute meaningfully to the institutional debate.

Beyond the interpretive ambiguities and operational challenges that will inevitably arise, the direction set by the 2025 draft bill—read in conjunction with the principles recently established by the Court of Cassation—extends beyond the mere possibility of relieving individual healthcare professionals of criminal liability in specific cases and sends a clear signal to healthcare organizations and their executives. It is no longer tenable to treat criminal negligence as a variable exclusively tied to individual professional error; preventive and proactive action becomes necessary, achievable also through the adoption of rigorous assessment tools for organizational and managerial risk.

In light of the analysis conducted, we consider it important to emphasize that, while the assessment of organizational negligence does not represent, strictly speaking, a new jurisprudential strand—a principle already partly consolidated by criminal and civil case law—the novelty lies rather in its proposed legislative codification. The new Article 590-septies, if approved, would constitute the first systematic provision aimed at linking medical negligence also to objective and systemic parameters.

Should the reform be approved, individual healthcare professionals could invoke, as a means of mitigating their own criminal liability, structural inadequacies and resource constraints, asking the court to assess their professional conduct in light of the structural context in which the error occurred. This shift, which could not be allowed to restrict the rights of the injured party, would also transfer a degree of liability toward the managerial and organizational sphere.

We believe that the redefinition of executive liability—considered beyond its strictly criminal law dimensions and approached with a constructive and proactive stance—could drive a genuine improvement and rethinking of the healthcare delivery model, one that moves beyond mere compliance obligations and repositions key instruments as central tools of clinical governance. In this context, the traceability of decision-making processes, the appropriate allocation of resources, and the formalization of safety protocols should no longer be confined to regulatory fulfilment but should instead serve as a fundamental objective benchmark for assessing the appropriateness of senior management functions.

The consolidation of a structured incident reporting system, the introduction of periodic organizational audits focused on high-risk areas, the implementation and monitoring of all appropriate preventive measures for the control of healthcare-associated infections, the traceability of managerial decision-making through documentary standards, the progressive integration of organizational models into a unified risk governance framework, targeted managerial training in risk governance, and the transition from a traditional approach to risk management toward an integrated Enterprise Risk Management (ERM) model may all come to constitute indispensable evidentiary obligations for healthcare organizations and their apex managers.

Interpreted in this proper perspective, the redefinition of executive liability may therefore represent the principal objective benchmark for measuring the exercise of managerial functions and an essential prerequisite for pursuing the safety of care and the constitutional protection of patients’ right to health.

Although not directly exportable to other legal systems, the Italian approach could contribute to the broader international debate on how to translate systems thinking about patient safety into enforceable institutional obligations.

## Figures and Tables

**Table 1:** Glossary of key Italian legal terms used in this article.

Legal Term	Italian Term	Operational Definition
Criminal Code	Codice penale (c.p.)	The Italian Criminal Code, enacted by Royal Decree No. 1398 of 19 October 1930 and subsequently amended. The primary source of substantive criminal law in Italy. Cited throughout this article as &quot;c.p.&quot; in references to individual provisions (e.g., Art. 40 c.p., Art. 590-sexies c.p., Art. 590-septies c.p.).
Negligence/Fault	Colpa	Absence of intent to cause a harmful event, occurring due to breach of a duty of prudence, diligence, or skill (Arts. 43 and 589–590 c.p.).
Lack of skill	Imperizia	Deficit of technical competence. Under Art. 590-sexies c.p., the only form of fault for which non-punishability may be invoked.
Negligence stricto sensu	Negligenza	Omission of precautions due in professional activity. Remains fully punishable under Art. 590-sexies c.p.
Imprudence	Imprudenza	Hasty, reckless, or insufficiently considered action. Remains fully punishable under Art. 590-sexies c.p.
Gross negligence	Colpa grave	Most severe degree of fault; blatant and inexcusable violation of diligence rules.
Slight negligence	Colpa lieve	Lesser degree of fault. Under DDL 2025, all forms classified as slight and guideline-compliant would be exempt from criminal liability.
Causal nexus	Nesso causale	Aetiological link between conduct and harmful event. In criminal proceedings: high logical probability (Franzese, No. 30328/2002).
Professional liability	Responsabilità professionale	Liability from negligent/unskilful professional service. In healthcare, governed by Law No. 24/2017.
Guarantor position	Posizione di garanzia	Legal duty to prevent harmful events by virtue of institutional role. Basis for criminal liability for improper omission (Art. 40(2) c.p.).
Criminal liability	Responsabilità penale	Strictly personal and blameworthy liability (Art. 27 Const.); requires objective and subjective elements.
Organizational fault	Colpa di organizzazione	Liability arising from structural, procedural, or governance deficits attributable to the organization. Central in proposed Art. 590-septies c.p.

## Data Availability

No new data were created or analyzed in this study. Data sharing is not applicable to this article.

## Abbreviations

The following abbreviations are used in this manuscript:

CAO	Chief Administrative Officer
CEO	Chief Executive Officer
CME	Continuing Medical Education
CMO	Chief Medical Officer
HAI	Healthcare-Associated Infections
HMD	Hospital Medical Director
HTA	Health Technology Assessment
SSN	Sistema Sanitario Nazionale (Italian National Service)
c.p.	Codice Penale (Italian Criminal Code)
